# Supplementary material for: The Role of Patient Expectations in Treatment Outcome and Satisfaction in Osteoarthritis: A Scoping and Mapping Review
Source: J Clin Med. 2025 Nov 28;14(23):8440. doi: 10.3390/jcm14238440 (PMC12693133; doi:10.3390/jcm14238440)
Supplement: Supplementary file 1 [file jcm-14-08440-s001.zip › jcm-3974041-supplementary.pdf]

Supplementary File S1. Search strategy.

PubMed

("Osteoarthritis"[Mesh] OR osteoarthrit*[tiab])

AND ("Expectation*"[tiab] OR "Patient Expectations"[Mesh] OR belief*[tiab] OR perception*[tiab] OR hope*[tiab] OR treatment outcome*[tiab])

AND (psychological[tiab] OR self-efficacy[tiab])

Embase

osteoarthrit*:ti,ab

AND ('expectation'/exp OR expectation*:ti,ab OR belief*:ti,ab OR perception*:ti,ab OR hope*:ti,ab)

AND (psychological factor*:ti,ab OR self-efficacy:ti,ab)

CINAHL

(MH "Osteoarthritis+" OR TI osteoarthrit* OR AB osteoarthrit*)

AND (TI expectation* OR AB expectation* OR MH "Patient Attitudes" OR TI belief* OR AB belief* OR TI perception* OR AB perception* OR TI hope* OR AB hope* OR TI "treatment outcome*" OR AB "treatment outcome*")

AND (TI psychological OR AB psychological OR TI "self-efficacy" OR AB "self-efficacy")

PsycINFO

(DE "Osteoarthritis" OR TI osteoarthrit* OR AB osteoarthrit*)

AND (TI expectation* OR AB expectation* OR DE "Patient Expectations" OR TI belief* OR AB belief* OR TI perception* OR AB perception* OR TI hope* OR AB hope* OR TI "treatment outcome*" OR AB "treatment outcome*")

AND (TI psychological OR AB psychological OR TI "self-efficacy" OR AB "self-efficacy")

Web of Science

TS=(osteoarthrit*)

AND TS=(expectation* OR belief* OR perception* OR hope* OR "treatment outcome*")

AND TS=(psychological OR "self-efficacy")
